# Co-Receptor CD8-Mediated Modulation of T-Cell Receptor Functional Sensitivity and Epitope Recognition Degeneracy

**DOI:** 10.3389/fimmu.2013.00329

**Published:** 2013-10-21

**Authors:** Barbara Szomolay, Tamsin Williams, Linda Wooldridge, Hugo Antonius van den Berg

**Affiliations:** ^1^University of Warwick, Coventry, UK; ^2^Institute of Infection and Immunity, Cardiff University School of Medicine, Cardiff, UK; ^3^Faculty of Medical and Veterinary Sciences, University of Bristol, Bristol, UK

**Keywords:** T-cell receptor, co-receptor CD8, degeneracy, functional sensitivity, ligand focusing, mathematical model

## Abstract

The interaction between T-cell receptors (TCRs) and peptide epitopes is highly degenerate: a TCR is capable of interacting productively with a wide range of different peptide ligands, involving not only cross-reactivity proper (similar epitopes elicit strong responses), but also polyspecificity (ligands with distinct physicochemical properties are capable of interacting with the TCR). Degeneracy does not gainsay the fact that TCR recognition is fundamentally specific: for the vast majority of ligands, the functional sensitivity of a given TCR is virtually null whereas this TCR has an appreciable functional sensitivity only for a minute fraction of all possible ligands. Degeneracy can be described mathematically as the probability that the functional sensitivity, of a given TCR to a randomly selected ligand, exceeds a set value. Variation of this value generates a statistical distribution that characterizes TCR degeneracy. This distribution can be modeled on the basis of a Gaussian distribution for the TCR/ligand dissociation energy. The kinetics of the TCR and the MHCI molecule can be used to transform this underlying Gaussian distribution into the observed distribution of functional sensitivity values. In the present paper, the model is extended by accounting explicitly for the kinetics of the interaction between the co-receptor and the MHCI molecule. We show that T-cells can modulate the level of degeneracy by varying the density of co-receptors on the cell surface. This could allow for an analog of avidity maturation during incipient T-cell responses.

## Introduction

Thymus-derived lymphocytes (T-cells) recognize peptide antigens via antigen-specific receptors (TCRs); in particular, CD8^+^ cytotoxic T lymphocytes (CTLs) recognize short peptides presented by major histocompatibility complex (MHC) class I molecules (1). Estimates of the human TCR diversity suggest that there are ∼10^8^ different antigen receptors in the naïve T-cell pool (2), which raises the question how such a limited TCR repertoire can provide effective immunity to perhaps over 10^15^ distinct pMHCs (2). The discrepancy suggests that even while TCRs are highly specific, a considerable degree of degeneracy remains (we use *degeneracy* as a term of art to cover both polyspecificity and cross-reactivity). The central importance of degeneracy was first pointed out by Mason (3) and later confirmed by others such as (4). Experimental and mathematical studies confirm that TCR recognition is highly degenerate: a single TCR may be able to recognize, at physiologically relevant degrees of functional sensitivity, over one million different peptides in the context of a single MHCI molecule (2, 5), an estimate that takes into account the binding specificity of the MHC molecule, but not the additional selection imposed by the stringency requirements of peptide cleavage and processing in the presentation pathway. The latter constitutes an epitope diversity filter that is instrumental in regulating the immuno-visibility of salient epitopes (6). The issue of whether there exists an optimal level of TCR repertoire diversity was reviewed by Nikolich-Žuglich et al. (7), and various authors have reviewed the functional repercussions at the systems level (8–10).

The interaction between TCR and pMHCI ligand can be modulated by the co-receptor CD8 in several ways: (i) promoting the association of TCR and pMHCI; (ii) stabilizing the TCR/pMHCI interaction; and (iii) enhancing the rate at which the TCR/CD3 complex attains signaling status by association of TCR/CD3 with protein tyrosine kinases such as p56^lck^ and adaptor molecules such as LAT and LIME (5, 11–16). The first of these three mechanisms modifies the affinity of the TCR/pMHCI interaction, whereas the third alters the time it takes for an engaged TCR/CD3 complex to attain full signalosome status. In particular, CD8 can enhance the TCR/pMHCI association rate by 50%, and reduce the TCR/pMHCI dissociation rate by at least 50% (16, 17), and CD8 modulates the rate of immune receptor tyrosine-based activation motif (ITAM) phosphorylation, by recruiting TCR/pMHCI complexes to membrane micro-domains at a rate which depends on the affinity of pMHCI/CD8 binding (16).

These findings suggest that CD8 not only controls degeneracy, but also differentially regulates functional sensitivity, that is, the T-cell can increase its sensitivity for one ligand, while reducing it for others. By varying the level of CD8 expression, the T-cell can increase its sensitivity to the disease-associated antigen, while at the same time decreasing its sensitivity to antigens associated with healthy conditions. This novel mode of co-receptor action could be critical in ensuring that the TCR repertoire retains the ability to respond to antigenic challenges, while avoiding autoimmunity.

T-cell antigen recognition can be expressed in terms of its *functional sensitivity* (18). One of the main determinants of functional sensitivity is the rate at which a single agonist copy elicits TCR triggering. Functional sensitivity depends on bio-molecular parameters such as the TCR/pMHCI on-rate and off-rate. The molecular kinetics at the T-cell:antigen-presenting cell (APC) interface determine this relationship. This kinetic theory resolves the long-standing controversy over whether T-cell activation is governed by affinity or off-rate (cf. (19, 20)); the theory shows that *both* parameters play a role, but in the so-called MHC-limited regime, the off-rate is the main governing factor. However, the on-rate and the off-rate together determine whether or not the kinetics is MHC-limited (21–23).

The aim of the present study is to explore the kinetic basis of the role of CD8 in regulating degeneracy and functional sensitivity. Our model generalizes the classical kinetic proofreading model, as proposed by McKeithan (24), and later modified by others (21, 22, 25–27). The kinetic proofreading model assumes that the TCR needs to remain bound to a pMHC in order for the TCR/CD3 complex to become fully activated, via several modification steps such as phosphorylation of several tyrosine residues on the TCR complex, and recruitment and subsequent activation of ZAP-70 (28).

The statistics of TCR degeneracy is modeled by relating the TCR/pMHCI mean interaction time with the dissociation energy according to Arrhenius theory. We show that by varying the total co-receptor density and key kinetic parameters, the T-cell can modulate the level of degeneracy. Furthermore, we compare our results with experimental data for HLA A^∗^0201 mutants with altered binding affinity for CD8.

## Theory: TCR/pMHCI/CD8 Kinetics and TCR Degeneracy

We assume (i) that the TCR/CD3 complex on the T-cell surface becomes triggered (achieves signalosome status) during an interaction with a pMHCI ligand if it undergoes *n* ITAM phosphorylations, where *n* is a positive integer; and (ii) that the presence of a CD8 molecule bound to the pMHCI ligand affects the TCR/pMHCI association and dissociation rates, as well as the rate at which the TCR/CD3 complex progresses through the phosphorylation sequence. The co-receptor CD8 is associated with TCR/CD3 with tyrosine kinases such as p56^lck^, which phosphorylates tyrosine residues within the ITAMs.

**Figure 1:**
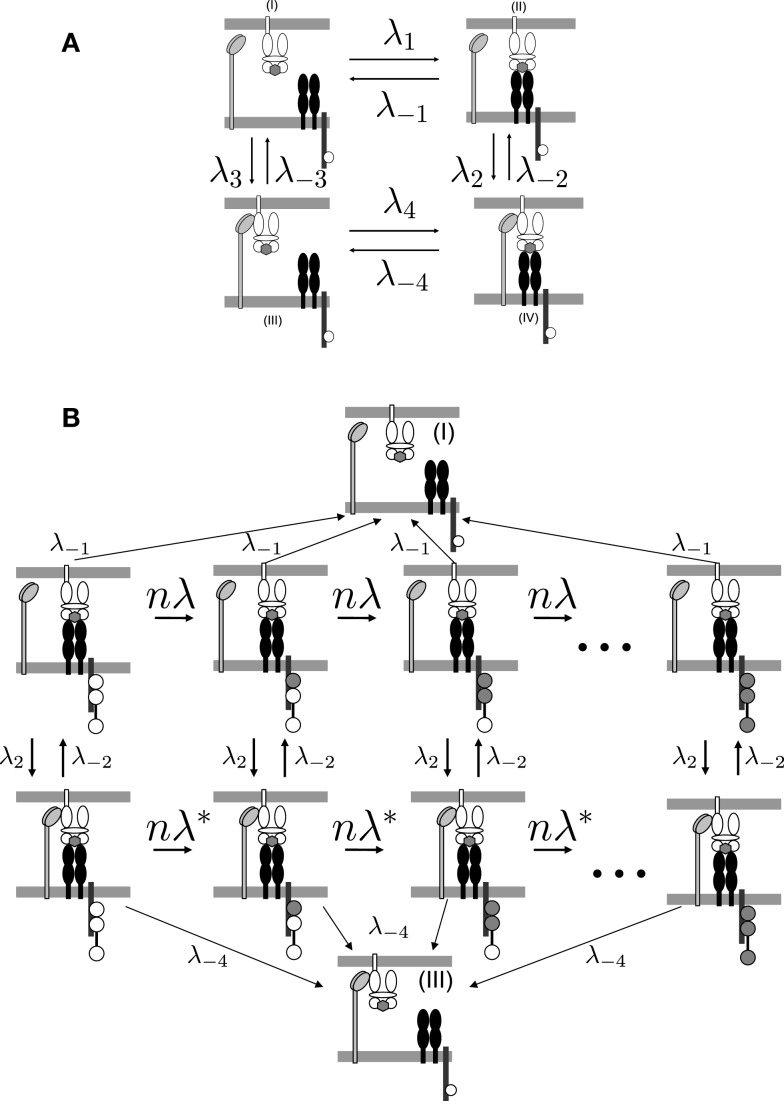
**TCR/pMHCI/CD8 interactions represented in the model**. **(A)** Kinetics of TCR/pMHCI/CD8 complex formation. TCRs and CD8s are located on the T-cell side and pMHCIs on the APC side. Four possible states are shown: in (I) and (II), pMHCI is not bound to CD8 whereas in (III) and (IV), pMHCI is bound to CD8; in (I) and (III), pMHCI is not bound to TCR/CD3 whereas in (II) and (IV), pMHCI is bound to TCR/CD3. Transition rates between these four states are indicated with forward rates labeled as λ*_i_* and backward rates labeled as λ_−_*_i_* (where *i* = 1, 2, 3, 4). The white circles in the tails of the TCR/CD3 complex represent the ITAMs, which are present on the cytoplasmic tails of the γ, δ, the two ϵ, and the two ζ polypeptide chains of the CD3 complex; only one such tail is indicated here, for clarity. There are *n* such ITAMs, with only three shown here for the sake of clarity. This part of the kinetic scheme is assumed to be in equilibrium. **(B)** The TCR/pMHCI bound states (II) and (IV) are further subdivided into *n* states each depending on the number of phosphorylated ITAMs, resulting in a Markov chain. The triggered states are the right-most ones where all *n* ITAMs have been phosphorylated (indicated as filled-in circles). At each state a reversal (at rate λ_−1_ or λ_−4_) to the unbound states (I) or (III), respectively, can occur. Transitions between the sequences occur at rates λ_2_ and λ_−2_. Left-to-right transitions within the sequences correspond to ITAM phosphorylation by kinases such as p56^lck^ and ZAP-70, at rate *n*λ = *n*/*T_R_* in the sequence with CD8 unbound and at rate $n\lambda^{*}=n∕T_{R}^{*}$ in the chain with CD8 bound. The Markov chain serves to calculate the TCR triggering probability and is not assumed to be in equilibrium.

To determine the kinetics of the TCR/pMHCI/CD8 interactions, consider the four binding states of a pMHCI molecule, labeled (I–IV) in Figure 1A. The states in which the TCR and the pMHCI ligand are bound can be further subdivided into different states according to the number of phosphorylated ITAMs, as shown in Figure 1B. The transition rate between these states is *n*/*T_R_*, where *T_R_* is the average time required to go through the entire sequence. The notation *T_R_* emphasizes that this quantity is the average time for the TCR/CD3 complex to become triggered. We call this the *TCR triggering threshold*. Typical values of TCR triggering threshold *T_R_* are in the range of 5–15 s (29).

### Kinetic equations

The model describes the kinetics of the interactions between the TCR, pMHCI molecules, and co-receptor CD8 in the contact area between a T-cell and an APC. This area is occupied by TCRs and CD8s on the T-cell side and by pMHCIs on the APC side, whereby CD8 binds pMHCI at a distinct site of the TCR/pMHCI complex. ITAMs are located on the ζ-chains of the CD3 complex associated with the TCR and are represented schematically in Figure 1B.

#### Interactions between TCR, pMHCI, and CD8

Key quantities in the model (summarized in Table 1) are the densities of free receptors on the T-cell and free ligands on the APC, which can also form complexes with the receptors. The surface densities of the TCR, CD8, and pMHCI molecules are subject to the following conservation laws:

(1) $$\begin{array}{ll} M_{T} & =M+M_{R}+M_{X}+M_{XR}\;, \end{array}$$

(2) $$\begin{array}{ll} X_{T} & =X+M_{X}+M_{XR}\;, \end{array}$$

(3) $$\begin{array}{ll} R_{T} & =R+M_{R}+M_{XR}\;, \end{array}$$

which state that for each species, the total density must equal the sum of all bound forms plus the free density. We define pseudo-unimolecular association rates λ*_i_*(*i* = 1, …, 4) as follows (see Figure 1A):

(4) $$\begin{array}{ll} \lambda_{1} & =\Lambda_{1}R,\;\lambda_{2}=\Lambda_{2}X, \end{array}$$

(5) $$\begin{array}{ll} \lambda_{3} & =\Lambda_{3}X,\;\lambda_{4}=\Lambda_{4}R, \end{array}$$

where Λ_1_ − Λ_4_ are the two-dimensional association rates (cm^2^s^−1^) for TCR/pMHCI or CD8/pMHCI binding. Two-dimensional dissociation constants (cm^−2^) are defined as follows:

(6) $$\begin{array}{ll} K_{1} & =\frac{\lambda_{-1}}{\lambda_{1}}R, \end{array}$$

(7) $$\begin{array}{ll} K_{2} & =\frac{\lambda_{-2}}{\lambda_{2}}X, \end{array}$$

(8) $$\begin{array}{ll} K_{3} & =\frac{\lambda_{-3}}{\lambda_{3}}X, \end{array}$$

(9) $$\begin{array}{ll} K_{4} & =\frac{\lambda_{-4}}{\lambda_{4}}R, \end{array}$$

**Table 1:** **Model parameters and variables**.

*M*	Free pMHCI density
*M_R_*	TCR/pMHCI density without CD8 bound
*M_X_*	pMHCI/CD8 density without TCR bound
*M_XR_*	TCR/pMHCI/CD8 density
*X*	Free CD8 density
*R*	Free TCR density
*M_T_*	Total pMHCI density
*X_T_*	Total CD8 density
*R_T_*	Total TCR density

In order for a system of reactions to be in thermal equilibrium, each individual reaction must be at equilibrium (the principle of detailed balance):

(10) $$\begin{array}{ccc} \lambda_{1}M & =\lambda_{-1}M_{R},\;\lambda_{2}M_{R}=\lambda_{-2}M_{XR}, & \\ \lambda_{3}M & =\lambda_{-3}M_{X},\;\lambda_{4}M_{X}=\lambda_{-4}M_{XR}, \end{array}$$

from which it follows that *K*_1_*K*_2_ = *K*_3_*K*_4_. Combining this with the conservation laws with we obtain:

(11) $$\begin{array}{ccc} M & =\frac{M_{T}}{C},\;M_{R}=\frac{M_{T}\lambda_{1}}{C\lambda_{-1}}, & \\ M_{X} & =\frac{M_{T}\lambda_{3}}{C\lambda_{-3}},\;M_{XR}=\frac{M_{T}\lambda_{1}\lambda_{2}}{C\lambda_{-1}\lambda_{-2}}, \end{array}$$

where

(12) $$C=1+\frac{\lambda_{1}}{\lambda_{-1}}+\frac{\lambda_{3}}{\lambda_{-3}}+\frac{\lambda_{1}\lambda_{2}}{\lambda_{-1}\lambda_{-2}}.$$

These results can also be expressed in terms of standard affinity constants:

(13) $$\begin{array}{ccc} M_{R} & =\frac{M_{T}R}{K_{1}+R+XK_{1}∕K_{3}+RX∕K_{2}}, & \\ M_{X} & =\frac{M_{T}X}{K_{3}+X+RK_{3}∕K_{1}+RX∕K_{4}},\\ M_{XR} & =\frac{M_{T}XR}{K_{1}K_{2}+RK_{2}+XK_{4}+RX}. \end{array}$$

The co-receptor CD8 modulates the rate of TCR triggering. Three major modulatory functions of the co-receptor have been documented: modulation of TCR/pMHCI on-rate, TCR/pMHCI off-rate, and of the ITAM phosphorylation rate. These effects can be represented by dimensionless multipliers.

(i) enhanced TCR/pMHCI on-rate:
  $$\Lambda_{4}=\gamma_{\text{on}}\Lambda_{1}\text{ where }\gamma_{\text{on}}\geq 1;$$
(ii) reduced TCR/pMHCI off-rate:
  $$\lambda_{-4}=\gamma_{\text{off}}\lambda_{-1}\text{ where }0<\gamma_{\text{off}}\leq 1;$$
(iii) increased phosphorylation rate, which is equivalent to a reduced TCR triggering threshold $T_{R}^{*}$
  (14) $$\lambda ={\gamma_{R}\lambda }^{*},\text{ where }\gamma_{\text{R}}\leq 1\text{ and }\lambda =\frac{1}{T_{R}}\text{ and }\lambda^{*}=\frac{1}{T_{R}^{*}}.$$

It is sometimes convenient to combine the on-rate and off-rate effects into a single coefficient, as follows:

(15) $$\gamma_{\text{kin}}=\frac{\gamma_{\text{off}}}{\gamma_{\text{on}}}=\frac{K_{4}}{K_{1}}=\frac{K_{2}}{K_{3}},\text{ where }0<\gamma_{\text{kin}}\leq 1.$$

From equation (15), we have γ_kin_ ≤ γ_off_. We rewrite *M_R_*, *M_X_*, and *M_XR_*, as given by equation (13), in terms of γ_kin_:

(16) $$\begin{array}{ccc} M_{R} & =M_{T}\frac{R∕K_{1}}{1+R∕K_{1}+X∕K_{3}+RX∕\left(K_{1}K_{3}\gamma_{\text{kin}}\right)}, & \\ M_{X} & =M_{T}\frac{X∕K_{3}}{1+R∕K_{1}+X∕K_{3}+RX∕\left(K_{1}K_{3}\gamma_{\text{kin}}\right)},\\ M_{XR} & =M_{T}\frac{RX∕\left(K_{1}K_{3}\right)}{\gamma_{\text{kin}}\left(1+X∕K_{3}+R∕K_{1}\right)+XK_{3}∕\left(RK_{1}\right)}. \end{array}$$

To non-dimensionalize this system, we introduce the following dimensionless quantities:

(17) $$\begin{array}{llll} x & =\frac{X}{K_{3}}\;,\;r=\frac{R}{K_{1}}\;,\;x_{T}=\frac{X_{T}}{K_{3}}\;,\;r_{T}=\frac{R_{T}}{K_{1}}\;,\; & & \;\\ \kappa & =\frac{K_{1}}{K_{3}}\;,\;m_{T}=\frac{M_{T}}{K_{1}}\;. \end{array}$$

It follows from equation (17) that *M_T_*/*K*_3_ = κ*m_T_*. By equations (16) and (17), we have the following non-linear system of equations that determines the surface densities of free and bound receptors of all species:

(18) $$\begin{array}{ccc} x_{T} & \;=\;x\;+\;\kappa m_{T}\frac{x}{1\;+\;x\;+\;r\;+\;xr∕\gamma_{\text{kin}}}+\kappa m_{T}\frac{xr}{\gamma_{\text{kin}}\left(1+x+r\right)+xr}, & \\ r_{T} & =r+m_{T}\frac{r}{1+x+r+xr∕\gamma_{\text{kin}}}+m_{T}\frac{xr}{\gamma_{\text{kin}}\left(1+x+r\right)+xr}. \end{array}$$

This system is readily solved numerically for *x* and *r*, given the parameters γ_kin_, κ, *r_T_*, and *m_T_*.

#### The TCR triggering rate

The functional sensitivity of the TCR is represented in the present model as the rate at which TCR/CD3 complexes attain signalosome status. To calculate this TCR triggering rate, which we shall denote by *W*, consider the Markov chain as depicted in Figure 1B, which is best thought of as a system of two coupled linear Markov chains. The TCR/CD3 complex has to undergo *n* phosphorylations for the TCR to be triggered. When the co-receptor is not bound to the TCR/pMHCI complex, individual phosphorylation steps proceed at rate *n*/*T_R_* ≡ *n*λ whereas when the co-receptor is engaged, this rate is $n∕T_{R}^{*}\equiv n\lambda^{*}\geq n\lambda$. The factor *n* arises simply as a matter of scaling, so that the average time to progress through the chain of phosphorylations equals *T_R_*.

In reality, signalosome formation involves several other types of event besides ITAM phosphorylation, such as binding of ZAP-70, engagement of LAT, and so on. To avoid cumbersome notation we shall formulate the model as if ITAM phosphorylation were the only type of event; the essential theory is not materially affected by this simplification. We do not assume an equilibrium state for the Markov chain: the complex starts at zero phosphorylations at the beginning of every encounter with a pMHCI ligand and proceeds forward stochastically.

The pMHCI/TCR/CD3 complex may not attain the *n*th state (triggered state), but instead the pMHCI/TCR engagement may terminate, which happens at rate λ_−1_ when the co-receptor is not bound and at rate λ_−4_ ≤ λ_−1_ when the co-receptor is engaged. We assume that upon TCR/pMHCI dissociation the CD3 complex reverts to the basal state of zero ITAM phosphorylations sufficiently rapidly that the TCR/CD3 complex will be in this completely unphosphorylated state when the next encounter with a pMHCI molecule occurs. Essentially, this means that the CD3 complex is more susceptible to the action of phosphorylases and/or less susceptible to the action of kinases when the TCR is not engaged. A mechanistic explanation underpinning this assumption lies outside the scope of the present model.

The probability that the TCR/CD3 complex will undergo another ITAM phosphorylation is given by

(19) $$P_{a}^{0}=\frac{n\lambda }{\lambda_{-1}+n\lambda +\lambda_{2}}$$

in the case where the co-receptor CD8 is not engaged and by

(20) $$P_{a}^{*}=\frac{n\lambda^{*}}{\lambda_{-4}+n\lambda^{*}+\lambda_{-2}}$$

when CD8 is engaged. The following expression gives the probability that the system switches from the chain with CD8 unbound to the chain with CD8 bound:

(21) $$P_{b}^{0}=\frac{\lambda_{2}}{\lambda_{-1}+n\lambda +\lambda_{2}}$$

whereas the system will switch from CD8-unbound to CD8-bound with probability

(22) $$P_{b}^{*}=\frac{\lambda_{-2}}{\lambda_{-4}+n\lambda^{*}+\lambda_{-2}}.$$

TCR triggering requires completion of all steps before the TCR/pMHCI complex comes apart. We shall find an expression for the probability that the CD3 complex will ultimately attain completion when starting from *i* completed steps. Let $P_{i}^{0}$ denote this probability for the case with CD8 unbound and $P_{i}^{*}$ for the case with CD8 bound. The TCR triggering probability is then found as $P_{0}^{0}$ if CD8 is unbound when pMHCI docks the TCR, and $P_{0}^{*}$ if CD8 is bound. These triggering probabilities allow us to calculate the TCR triggering rate *W*. In particular, *W* can be expressed as the rate at which TCR/pMHCI complexes dissociate, times the probability that whenever a given TCR/pMHCI docking commences, the CD3 complex is ultimately triggered (the probabilities $P_{0}^{0}$ and $P_{0}^{*}$ ). In formula, this statement is represented as follows:

(23) $$W=M\lambda_{1}P_{0}^{0}+M_{X}\lambda_{4}P_{0}^{*}$$

which, by the principle of detailed balance, can be rewritten in terms of *dissociation rates*, as follows:

(24) $$W=R\lambda_{-1}P_{0}^{0}+M_{XR}\lambda_{-4}P_{0}^{*}.$$

The law of total probability yields the following system of coupled difference equations:

(25) $$\left[\begin{array}{c} P_{i-1}^{0}\\ P_{i-1}^{*} \end{array}\right]=\frac{1}{1-P_{b}^{0}P_{b}^{*}}\left[\begin{array}{cc} P_{a}^{0} & P_{b}^{0}P_{a}^{*}\\ P_{b}^{*}P_{a}^{0} & P_{a}^{*} \end{array}\right]\left[\begin{array}{c} P_{i}^{0}\\ P_{i}^{*} \end{array}\right]$$

which can be solved to give

(26) $$\left[\begin{array}{c} P_{0}^{0}\\ P_{0}^{*} \end{array}\right]=\frac{1}{{\left(1-P_{b}^{0}P_{b}^{*}\right)}^{n}}{\left[\begin{array}{cc} P_{a}^{0} & P_{b}^{0}P_{a}^{*}\\ P_{b}^{*}P_{a}^{0} & P_{a}^{*} \end{array}\right]}^{n}\left[\begin{array}{c} 1\\ 1 \end{array}\right]$$

where we have used the boundary condition

(27) $$P_{n}^{0}=P_{n}^{*}=1\;.$$

This boundary condition expresses the basic assumption that triggering is attained when the sequence has been completed. To render the equations dimensionless, we introduce the following parameters:

(28) $$\nu =\frac{\Lambda_{2}K_{3}}{\lambda },\alpha =\frac{\lambda_{-1}}{\lambda },\delta =\frac{\lambda_{-2}}{\lambda }.$$

The scaled (dimensionless) TCR triggering rate is then given by the following expression:

(29) $$w=\alpha \left(\epsilon P_{0}^{0}+\zeta \gamma_{\text{off}}P_{0}^{*}\right)$$

where

(30) $$\begin{array}{ll} w & =\frac{W}{K_{1}m_{T}\lambda }; \end{array}$$

(31) $$\begin{array}{ll} \varepsilon & =\frac{r}{1+x+r+xr∕\gamma_{\text{kin}}}; \end{array}$$

(32) $$\begin{array}{ll} \zeta & =\frac{xr}{\gamma_{\text{kin}}\left(1+x+r\right)+xr}. \end{array}$$

The scaled TCR triggering rate *w* depends on ten dimensionless parameters (Table 2).

**Table 2:** **Dimensionless (scaled) parameters that govern functional sensitivity**.

*m_T_*	Scaled total pMHCI density
*x_T_*	Scaled total CD8 density
*r_T_*	Scaled total TCR density
α	Scaled TCR/pMHCI off-rate without CD8 bound
δ	Scaled pMHCI/CD8 off-rate with TCR bound
ν	Scaled kinetic effect of pMHCI/CD8 interactions with and without TCR
κ	Ratio of dissociation constants *K*_1_ and *K*_3_
γ_off_	Factor by which CD8 modulates TCR/pMHCI off-rate
γ_kin_	Factor by which CD8 modulates the TCR/pMHCI affinity
γ*_R_*	Factor by which CD8 modulates the TCR triggering threshold

### Statistical formulation of TCR degeneracy

To express TCR degeneracy mathematically, we consider the distribution of the triggering rate over the set of peptide ligands. This is just the set of *w_ij_*-values for a given TCR clonotype *i* over pMHCI ligands *j*. The distribution can be represented by plotting the probability ℙ(*w_ij_* > ω) as a function of ω. Such a graph shows how many randomly selected peptides would have triggering rate *w* larger than a set value ω. Let *T_ij_* denote the mean dwell time of the TCR/pMHCI interaction for TCR clonotype *i* and pMHCI species *j* so that $T_{ij}^{-1}$ is the TCR/pMHCI off-rate λ_−1_ (we have thus far suppressed subscripts for clone *i* and ligand *j* to keep notation uncluttered). Arrhenius theory furnishes the following relationship with the dissociation energy barrier Δ*U_ij_*:

(33) $$T_{ij}=T_{0}\;\exp\;\left(\Delta U_{ij}\right),$$

where *T*_0_ is the frequency factor and Δ*U_ij_* is expressed in Boltzmann units. We assume that the dissociation energy barrier arises as a result of a large number of individual reaction steps at the TCR/pMHCI interface. If these combine additively, then the Central Limit Theorem implies that Δ*U_ij_* has a Gaussian distribution. Letting *u_ij_* = Δ*U_ij_* − ln{*T_R_/T*_0_} we have *u_ij_* ∼ *N*(−μ, σ^2^), where μ > 0 and σ are the underlying parameters of the normal distribution *N*. The assumption that the mean is negative is a consequence of the fact that for the vast majority of TCR/pMHCI complexes the mean dwell time is less than the typical time required to complete the chain of ITAM phosphorylations. It now follows that the dimensionless parameter α, associated with the TCR/pMHCI off-rate, is log-normally distributed.

## Results

We investigated the effect of variations of the total CD8 density on the functional sensitivity of hypothetical ligands with various TCR/pMHCI off-rates. All variables and parameters are dimensionless (scaled) in the model simulations. The scaled parameters are summarized in Table 2 and their scaling is defined in equations (14), (15), (17), and (28).

### Ligand focusing and CD8-mediated control of degeneracy

**Figure 2:**
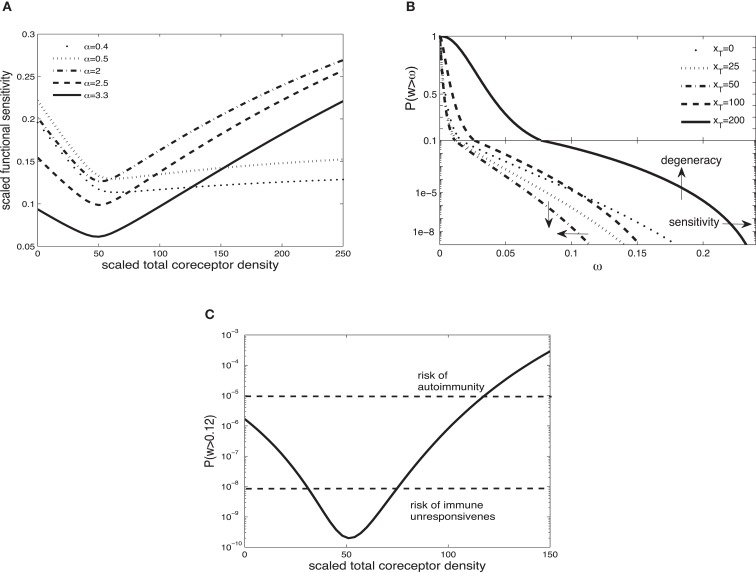
**(A)** Scaled functional sensitivity *w* as a function of scaled total CD8 density *x_T_* for various scaled TCR/pMHCI off-rates α. **(B)** Degeneracy curves ℙ(*w* > ω) for various scaled total CD8 density *x_T_*. **(C)** The probability ℙ(*w* > ω) as a function of CD8 density *x_T_*, at a set value of functional sensitivity ω = 0.12. The operating range of the probability ℙ is shown as a function of *x_T_* with dashed lines at ℙ(*w* > 0.12) = 10^−8^ and ℙ(*w* > 0.12) = 10^−5^. Parameter values: δ = 300, ν = 0.5, *n* = 100, γ_kin_ = 0.5, γ_off_ = 0.5, γ*_R_* = 0.3, κ = 5, *m_T_* = 10, *r_T_* = 10. The log-normal distribution has mean 2 and SD 0.2.

Figure 2A shows the scaled functional sensitivity *w* as a function of scaled total CD8 density *x_T_* with different scaled TCR/pMHCI off-rates α. A striking feature is the dip at *x_T_* ≈ 50, suggesting that at this CD8 density the T-cell is minimally responsive. As the scaled total CD8 density increases above *x_T_* ≈ 50, the scaled functional sensitivity increases for all hypothetical ligands.

It can be observed that a ligand with α = 2.5 (solid line) and a ligand with α = 0.5 (dotted line) show opposing changes in the scaled functional sensitivity: a ligand that is less potent at low CD8 becomes more potent at high CD8 and *vice versa*. Hence, changes in CD8 expression levels can differentially affect the potency of ligands, each of which is potentially a strong agonist. In effect, the T-cell can tune in on a specific ligand and thus control ligand promiscuity. We call this the principle of *ligand focusing*.

The corresponding degeneracy curves ℙ(*w* > ω) for different scaled total CD8 densities are shown in Figure 2B. The effect of the increase in co-receptor density on the sensitivity and degeneracy is indicated: sensitivity is a change in the horizontal direction (modulating the triggering threshold) and degeneracy is a change in the vertical direction (controlling cross-reactivity). As the scaled total CD8 density increases up to *x_T_* ≈ 50, the degeneracy curves *P*(*w* > ω) move to the left from the curve without CD8 (*x_T_* = 0): degeneracy and sensitivity decrease. By contrast, as the scaled total CD8 density increases above *x_T_* ≈ 50, the degeneracy curves move to the right, which means that degeneracy and sensitivity both increase. The overall effect is that the T-cell becomes more degenerate as CD8 levels increase. Thus, in addition to the focusing effect, the co-receptor also governs the overall degeneracy of the T-cell.

A high degree of degeneracy can increase the risk autoimmune disease. On the other hand, too low a degree of degeneracy could compromise the immune system’s ability to mount a timely and efficient response. To analyze these risks, suppose that the T-cell is activated if its integrated TCR triggering rate exceeds a certain value, termed *cellular activation threshold*. A simple model is to assume that the T-cell is activated if

(34) $$Z_{j}T_{I}w_{ij}>W_{\text{act}}$$

where *Z_j_* is the presentation level of ligand *j*, *T_I_* is the duration of the T-cell:APC interaction, and *W*_act_ is the activation threshold. For a given *Z_j_* and *T_I_*, there is a critical *w_ij_* which is the minimum value required to satisfy equation (34). Suppose for instance that this corresponds to *w_ij_* = 0.12. The corresponding probabilities ℙ(*w* > 0.12) are plotted in Figure 2C. Given the estimates of the TCR repertoire size, normal immune function is probably confined to an operating range of probabilities 10^−8^ to 10^−5^. Figure 2C shows how the level of CD8 can regulate the responsiveness to remain within this band; if the activation probability drops below this range, the risk of not responding to a pathogen looms, whereas at much elevated activation probabilities, the risk of autoimmunity is heightened.

### Modes of CD8-mediated modulation of functional sensitivity

**Figure 3:**
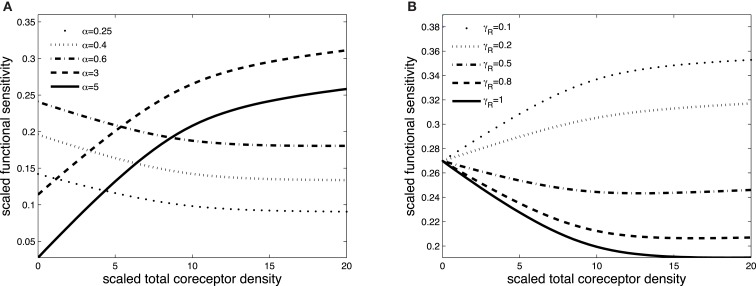
**Scaled functional sensitivity *w* as a function of scaled total CD8 density *x_T_***. **(A)** Curves for various values of the scaled TCR/pMHCI off-rate α. **(B)** Curves for various values of the factor γ*_R_* by which CD8 modulates the TCR triggering threshold. Parameter values in **(A,B)**: δ = 2.5, ν = 0.05, *n* = 100, γ_kin_ = 0.5, γ_off_ = 0.5, γ*_R_* = 0.5, κ = 1, *m_T_* = 10, *r_T_* = 10, α = 1.

**Figure 4:**
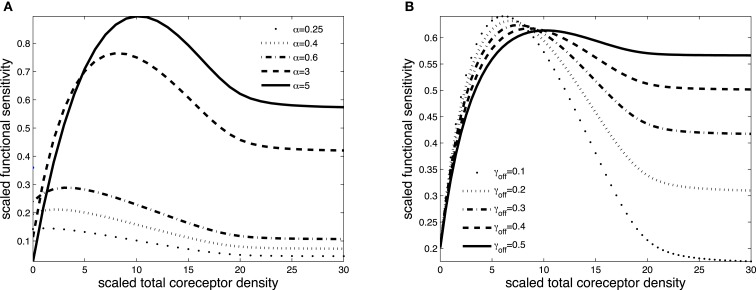
**Scaled functional sensitivity *w* as a function of scaled total CD8 density *x_T_***. **(A)** Curves for various values of the scaled TCR/pMHCI off-rate α. **(B)** Curves for various values of the dimensionless factor γ_off_ expressing the modulatory effect of CD8 on the TCR/pMHCI off-rate. Parameter values in **(A,B)** δ = 3, ν = 300, *n* = 100, γ_kin_ = 0.05, γ_off_ = 0.2, γ*_R_* = 0.5, κ = 2, *m_T_* = 10, *r_T_* = 10, α = 2.

Figures 3 and 4 exhibit the co-receptor effect on functional sensitivity in two parameter scenarios, where we assume a weak (ν = 0.05) or a strong (ν = 300) kinetic effect of pMHCI/CD8 interactions, respectively. When the kinetic parameter ν is small, increasing the levels of CD8 on the T-cell surface leads to enhanced functional sensitivity of ligands with α > 1, as shown in Figure 3A. By contrast, ligands with low off-rates (α < 1) become less potent when CD8 levels are increased. These opposite effects demonstrate CD8-mediated focusing on particular ligands.

Figure 3B shows the effect of increasing CD8 levels on a ligand that is optimal in the absence of CD8. When the modulatory effect of CD8 on the TCR triggering threshold is modest (γ*_R_* is near 1), the ligand becomes less potent as levels of CD8 are increased. On the other hand, when CD8 has a strong effect on the TCR triggering threshold (low γ*_R_*, since the time required to trigger the TCR/CD3 complex is shortened), increasing the levels of CD8 on the cell surface enhances the functional sensitivity to the ligand.

The effect of CD8 levels on functional sensitivity is shown in Figure 4A. For each ligand, there is an optimal CD8 level which depends on that ligand’s TCR/pMHCI off-rate α. This shows that the T-cell can favor the signaling strength of a given ligand by adjusting the density of CD8 molecules on its surface. As one would expect, the modulatory effect is most profound when the relative effect that the co-receptor exerts on the TCR/pMHCI off-rate is greatest; this is shown in Figure 4B. When CD8 alters the off-rate strongly, the functional sensitivity is strongly depressed when CD8 levels are increased beyond the optimal level, whereas for a moderate value of the modulatory multiplier γ_off_, the functional sensitivity remains at near-optimal levels when CD8 levels are increased. The value of γ_off_ may be expected to be different for different TCR/ligand combinations. In particular, when CD8 makes a substantial contribution to the binding energy, the multiplier γ_off_ will be low, and the co-receptor role in governing ligand optimality will be more pronounced.

### Co-receptor modulation of degeneracy via altered pMHCI/CD8 binding affinity

Wooldridge et al. (5, 30, 31) have shown that (i) increased pMHCI/CD8 interaction results in enhanced recognition of pMHCI by cytotoxic T-cells and (ii) increased pMHCI/CD8 interaction impairs pMHCI recognition specificity, suggesting that the pMHCI-CD8 interaction is essential in regulating the balance between optimal T-cell cross-reactivity and T-cell antigen specificity (32). These findings suggest that an optimal pMHCI/CD8 strength exists that yields maximum pMHCI sensitivity without loss of specificity. Motivated by these results, we consider a hypothetical scenario in which pMHCI mutant molecules with altered binding affinity for CD8 modulate TCR degeneracy.

In keeping with (17), the following pMHCI mutants are considered: A245V representing weak pMHCI/CD8 affinity (*K_D_* = 498 μM), wild-type (*K_D_* = 137.1 μM), Q115E representing slightly enhanced affinity (*K_D_* = 97.94 μM), and A2/α3k^b^ with enhanced affinity (*K_D_* = 10.87 μM). These values are based on Surface Plasmon Resonance experiments and should be regarded as “three-dimensional,” relating to the ligands in solution. However, the TCR/pMHCI interaction takes place in the “two-dimensional” environment of the T-cell:contact area, which essentially reduces spatial degrees of freedom of molecular motion and, moreover, introduces dynamics related to the forces that constrain the molecules to this environment, such as rotations with respect to the membrane plane, membrane fluctuations, and the translational motion of the membranes themselves (33). Furthermore, cooperativity interactions, such as the involvement of the co-receptor, may be profoundly altered. As a result, the rate constants can be markedly different in the “two-dimensional” environment; in particular, two-dimensional dissociation rates can be substantially faster (28). The ratio between the three- and two-dimensional affinities is a length measure, denoted *h* and called the confinement length (34, 35). Wu et al. (33) demonstrated that *h* is proportional to the range of motion available to the free forms of the interacting ligands along the spatial axis perpendicular to the two parallel membranes. Thus the inter-membrane separation distance provides an upper bound, and if the ranges of motion are broadly comparable, we may assume that the confinement length is roughly the same for all mutants involved.

**Figure 5:**
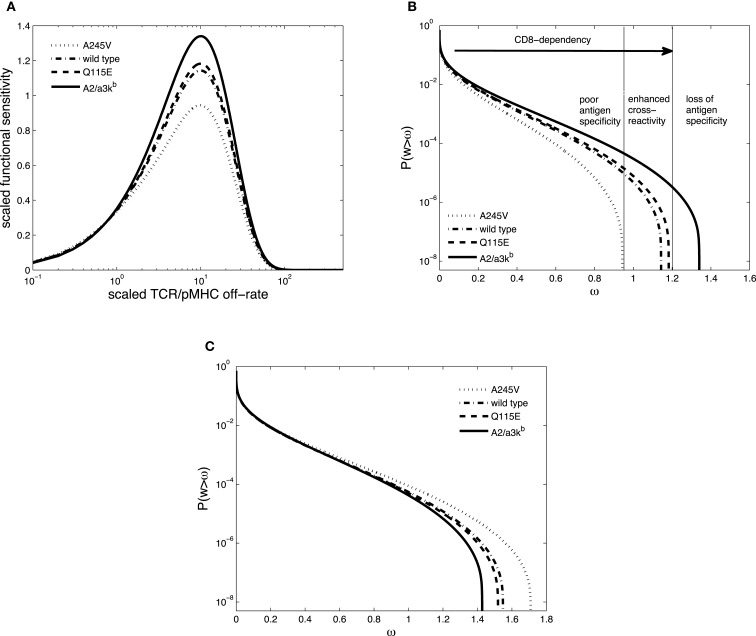
**(A)** Scaled functional sensitivity *w* as a function of scaled TCR/pMHCI off-rate α. **(B)** Degeneracy curves ℙ(*w* > ω) for HLA A^∗^0201 mutants with altered binding affinity for CD8: A245V (dotted line), wild-type (semi-dashed line), Q115E (dashed line), and A2/α3k^b^ (solid line). The three regions represent the overall pattern of CD8^+^ T-cell antigen specificity and the arrow indicates the strength of pMHCI/CD8 interaction. **(C)** Degeneracy curves ℙ(*w* > ω) for HLA A^∗^0201 mutants with altered binding affinity for CD8. The parameter values are the same as in A except for ν = 10. Parameter values in **(A,B)**: δ = 0.2, ν = 0.05, *n* = 100, γ_kin_ = 0.5, γ_off_ = 0.5, γ*_R_* = 0.2, κ = 1, *m_T_* = 10, *r_T_* = 10, *x_T_* = 10. The log-normal distribution has mean 5 and SD 0.5.

The two-dimensional dissociation constant for pMHCI/CD8 interaction without TCR bound, *K*_3_, appears in the scaled parameters κ, *x_T_*, and ν. Hence, by taking *K*_3_ to be proportional to *K_D_* for a given pMHCI mutant, we can simulate the impact of the altered pMHC/CD8 binding affinity on TCR degeneracy and sensitivity. Figure 5A shows the scaled functional sensitivity *w* as a function of scaled TCR/pMHCI off-rate α for the four hypothetical pMHCI mutants.

The co-receptor CD8 can modulate the specificity of antigen recognition, as shown in Figure 5B. Each TCR degeneracy curve corresponds to a given pMHCI mutant, where the one with the strongest pMHCI/CD8 binding affinity (A2/α3k^b^) is the most degenerate, with the largest antigen sensitivity. The three regions are a schematic representation of the overall pattern of CD8^+^ T-cell antigen specificity, as defined by Cole et al. (32). With increasing strength of pMHCI/CD8 affinity, as indicated by the arrow, the recognition efficiency of partially CD8-dependent ligands is enhanced and the spectrum of CD8^+^ T-cell antigen degeneracy becomes wider. Enhancing the kinetic effect of pMHCI/CD8 interactions (setting ν ≫ 1) results in the reversed pattern, as shown in Figure 5C.

Whereas in the MHC-limited kinetic regime, the behavior is as shown in Figure 5B, the degeneracy curves for the various mutants overlap in the TCR-limited regime. This suggests that excess levels of ligand, relative to the available levels of TCR molecules, can diminish the importance of the interaction between pMHCI and CD8. In principle, this endows the APC with a means to “override” the ligand focusing exerted by the T-cell, allowing a professional APC, such as a dendritic cell, to force a naïve T-cell, with which it has conjugated, to be maximally degenerate.

## Discussion

The co-receptors CD4 and CD8 are glycoproteins that modulate the interactions of the TCR with pMHCI and pMHCII molecules, by binding to invariant sites on these molecules (10). It is well-established that the co-receptors differentially regulate the responsiveness of the TCR to the ligand and thereby modulate TCR specificity (1). In particular, CD8 is known to affect both the on-rate and the off-rate of the TCR/pMHCI interaction (17, 36). This allows the co-receptor to differentially regulate the strengths of the various *potentially* strong agonists of the TCR. This accords with the finding that the strength of pMHCI/CD8 interaction is a determinant of T-cell degeneracy (5). This ligand focusing effect remains to be observed experimentally, to the best of our knowledge. Perhaps this is only to be expected inasmuch as the experimenter has to search for ligands that are sub-optimal under standard conditions but become better or worse agonists when CD8 levels are manipulated. Research is presently underway to identify such ligands and we anticipate that the phenomenon will be confirmed and eventually emerge as a pervasive “design principle” of cellular adaptive immunity.

Co-receptor-directed ligand focusing may allow the T-cell response to an antigen challenge to undergo an adaptive evolution that would be functionally analogous to affinity maturation in B-cell immunity. Moreover, CD8 modulation could allow for an elevated degeneracy among the earliest responding clones. This would ensure that at least one or more responding clones are activated sufficiently early in the course of the infection. Moreover, a gradual restriction of the degeneracy, coupled with an increase in functional sensitivity to the salient epitope, would then reduce the degeneracy of the response, which would gradually evolve from oligoclonal to one that is dominated by an optimally tuned single clone.

Disrupting the pMHCI/CD8 interaction impairs the ability of T-cells to recognize antigens. In particular, T-cell activation can be abrogated if the pMHCI/CD8 interaction is blocked (32), whereas increases in pMHCI/CD8 affinity have the opposite effect (37, 38). The contribution of CD8 to increase functional sensitivity appears to be crucial for weaker agonists (36). A comprehensive evaluation of clonal CD8^+^ T-cell degeneracy using combinatorial peptide libraries and APCs expressing mutant HLA A^∗^0201 molecules with altered pMHCI/CD8 affinity has shown that the co-receptor enhances T-cell degeneracy by increasing the range of agonist ligands that can elicit T-cell activation (5). Furthermore, increasing the affinity of CD8 for HLA A^∗^0201 by at least an order of magnitude resulted in the loss of cognate antigen specificity (5, 31). The affinity of the pMHCI/CD8 interaction may be directly linked to TCR degeneracy: increased pMHCI/CD8 interaction enhances CD8^+^ T-cell antigen sensitivity, but reduces CD8^+^ T-cell antigen specificity (32). This agrees with our main finding that variation of the co-receptor effect regulates the degree of T-cell degeneracy and antigen specificity.

A cornerstone of the present theory is that a certain amount of degeneracy is unavoidable, in view of the vast universe of possible peptides and the relatively modest number of TCR clonotypes that even a large mammal might be able to maintain in its standing repertoire. Moreover, salient epitopes, those associated with a disease state, and non-salient ones, such as self-peptides for which immune tolerance is required, will of necessity be “finely interleaved” subsets of the peptide universe (a mathematician would say that one subset is “dense” in the other), lest the tolerant subset forms a target for the rapidly evolving pathogens: the system cannot work if molecular mimicry is readily attained. From these two premises, it follows that a TCR must be degenerate, and also that this degeneracy must be susceptible to exquisite modulation. Against this line of reasoning a case could be made that the size of the ligand universe is effectively much smaller. For instance, if one considers *n*-mer peptides that are anchored to the MHC binding groove at *a* positions, and the region of the TCR that interacts with the peptide (roughly speaking, the CDR3 loop) makes contacts with *c* of the amino acid residues in the *m*-mer (so that 0 < *m* ≤ *n* − *a*), then there are 20*^m^*(*n* − *a*)!/(*m*!(*n* − *a* − *m*)!) effectively distinct pMHCI ligands as seen by the TCR. To give an extreme example, with *n* = 9, *a* = 2, and *m* = 1, this works out as just 20 × 7 = 140 distinct ligands. Perhaps the estimate *m* = 4 is more realistic: this gives only 20^4^ × 35 = 5.6 × 10^6^ functionally distinct ligands, which is of the same order as the TCR repertoire size. Whereas there may be some merit to this argument, its underlying image, essentially of CDR3 as a tape recorder head that interacts with only *m* amino acid residues and is indifferent to the *n* − *m* other ones, is a gross oversimplification. The physical behavior of the *m* amino acid residues at the contact sites cannot fail to be influenced by the *n* − *m* remaining ones (including the *a* anchor residues). This is overwhelmingly apparent not just from the basic principles of molecular dynamics, but also from the typical results obtained with combinatorial peptide library scans. A case in point is the finding that changes at the anchor position result in changes in the center of the peptide and therefore influence TCR binding (39, 40).

The present model indicates that intermediate levels of CD8 are associated with the lowest functional sensitivity. This suggests the following mechanism to maintain quiescent (naïve) T-cells in a relatively unresponsive state. When the T-cell receives the appropriate stimuli, it either up-regulates or down-regulates the co-receptor and a specific subset of its potential agonists “comes into focus.” Such signals are known to be transmitted via cytokine profiles in the T-cell’s surroundings (15) as well as costimulatory receptor-mediated signals transmitted by professional APCs (41). When no harm is detected, the default response of the naïve T-cells would be to “de-tune” whenever a strong signal is registered. Detuning of T-cells via alterations of CD8 expression levels, under control of cytokine stimuli, has been reported (42) and the connection between functional sensitivity, tolerance, and CD8 expression levels is well-established (14, 43, 44). On the other hand, when harm is detected and transmitted via a pro-inflammatory cytokine profile, the T-cell’s response would invert and the tendency would become to “tune in” to any supra-threshold stimulation. On a molecular-cellular level, this would involve a scanning mechanism whereby the stimulus would make the cell enter a mode in which it gradually alters the CD8 expression level whilst the received TCR signal (which will shift in magnitude as the CD8 level changes) feeds back onto this pathway. To the best of our knowledge, the molecular details of such a regulatory pathway have not been elucidated to date. However, we believe that it is well within the regulatory capabilities of cellular signaling networks; we have previously discussed similar mechanisms in more depth (45, 46).

Whilst the model includes the key components of TCR triggering, many important aspects have been omitted. In particular, we have neglected the spatial dynamics of TCR, CD8, and pMHCI within the immunological synapse, where the relative concentrations of p56^lck^ and CD45 will determine how quickly partially phosphorylated TCR/CD3 will reset to the basic state (47). A functional consequence of the exclusion of phosphorylases, for instance, could be the ability of the TCR/CD3 complex to be triggered over subsequent interactions with ligands that would in a normal context only have weak functional sensitivity.

In addition to kinetics of the interaction between TCR, MHCI, and CD8, we have only considered ITAM phosphorylation steps. It is well known that phosphorylated ITAMs orchestrate the activation of the Src-related protein tyrosine kinases which initiate TCR signaling. These kinases induce tyrosine phosphorylation of several polypeptides, including the transmembrane adaptors. Protein tyrosine phosphorylation subsequently leads to the activation of multiple pathways such as ERK, NF-κB, and NFAT (48, 49). Moreover, negative regulation of TCR signaling is key to avoiding hyper-activation. Notwithstanding the additional layers of complexity which our simple model ignores, we believe that the system of two linked proofreading chains as presented here does capture, in a qualitative sense, the essence of TCR triggering.

In summary, the present findings suggest that the co-receptor CD8 can differentially modulate functional sensitivity to its potential agonists, thereby modulating TCR degeneracy in a tunable fashion. The ligand focusing mechanism would allow each T-cell to have a wide range of potential agonists, even while only one of these would be a ligand of high functional sensitivity at any particular moment in time.

## Conflict of Interest Statement

The authors declare that the research was conducted in the absence of any commercial or financial relationships that could be construed as a potential conflict of interest.
