# Expression of Concern: Co-Receptor CD8-Mediated Modulation of T-Cell Receptor Functional Sensitivity and Epitope Recognition Degeneracy

**DOI:** 10.3389/fimmu.2014.00401

**Published:** 2014-08-18

**Authors:** Frontiers in Immunology Editorial Office

**Affiliations:** ^1^Frontiers in Immunology Editorial Office, Frontiers, Lausanne, Switzerland

## Expression of Concern

The Editorial Board has been informed by the authors of “Co-receptor CD8-mediated modulation of T-cell receptor functional sensitivity and epitope recognition degeneracy” that the parameter values as reported in the legends are mutually inconsistent and therefore the results of the simulations have to be reconfirmed. This is to notify readers that the data as published in the original article are not validated and that the authors have stated to the Editorial Board that a Corrigendum is in preparation.

