# Corrigendum: Co-Receptor CD8-Mediated Modulation of T-Cell Receptor Functional Sensitivity and Epitope Recognition Degeneracy

**DOI:** 10.3389/fimmu.2014.00443

**Published:** 2014-09-16

**Authors:** Barbara Szomolay, Tamsin Williams, Linda Wooldridge, Hugo Antonius van den Berg

**Affiliations:** ^1^University of Warwick, Coventry, UK; ^2^Institute of Infection and Immunity, Cardiff University School of Medicine, Cardiff, UK; ^3^Faculty of Medical and Veterinary Sciences, University of Bristol, Bristol, UK

**Keywords:** corrigendum, T cell, T cell receptor, T cell repertoire, T cell response

It has been kindly pointed out to us by Dr. Omer Dushek of Oxford University that the thermodynamic constraints (arising from the principle of detailed balance) impose the following condition on the parameters:

(1) $$\nu =\gamma_{\mathrm{kin}}\delta$$

which means that the parameter ν is fixed once γ_kin_ and δ have each been assigned a value. The objective of the paper was to exhibit the range of qualitative behaviors that is possible when pMHCI/CD8 kinetics interacts with TCR/pMHCI kinetics and to show how varying levels of the co-receptor at the T-cell surface may be able to modulate the functional sensitivity of the T-cell to various ligands in a differential fashion. These qualitative phenomena remain very much the same when we choose parameter values that respect the constraint ν = γ_kin_δ, as shown in the corrected figures that follow below (Figures 2–4). It is these qualitative patterns that are currently guiding experimental research to elucidate CD8-mediated ligand focusing in the T-cell system. The main thrust of the paper is therefore unaltered.

**Figure 2:**
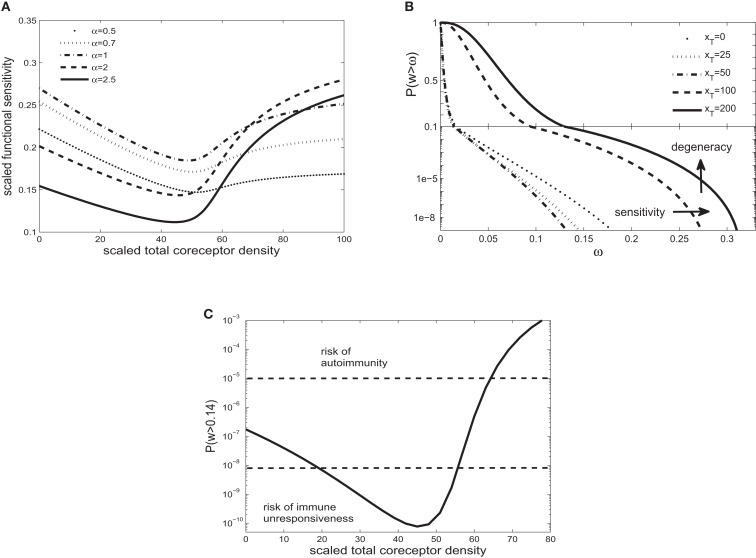
**(A)** Scaled functional sensitivity *w* as a function of scaled total CD8 density *x_T_* for various scaled TCR/pMHCI off-rates *α*. **(B)** Degeneracy curves ℙ(w > ω) for various scaled total CD8 density *x_T_*. **(C)** The probability ℙ(w > ω) as a function of CD8 density *x_T_*, at a set value of functional sensitivity ω = 0.14. The operating range of the probability ℙ is shown as a function of *x_T_* with dashed lines at ℙ(w > 0.14) = 10^−8^ and ℙ(w > 0.14) = 10^−5^. Parameters are as follows: $\delta =300,n=100,\gamma_{\mathrm{kin}}=0.1,\gamma_{\mathrm{off}}=0.5,\gamma_{R}=1,\kappa =5.5,m_{T}=10,r_{T}=10$. The log-normal distribution has mean 2 and SD 0.2. Changed: ω = 0.14, γ_kin_, γ_R_,κ and α’s in **(A)**.

**Figure 3:**
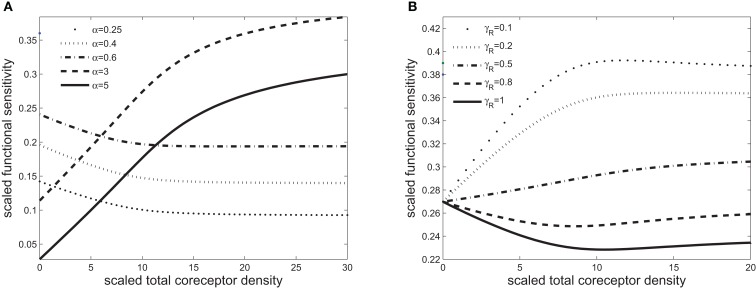
**Scaled functional sensitivity *w* as a function of scaled total CD8 density *x*_T_**. **(A)** Curves for various values of the scaled TCR/pMHCI off-rate α. **(B)** Curves for various values of the factor γ_R_ by which CD8 modulates the TCR triggering threshold. Parameters, unless stated otherwise, are as follows: δ = 2. 5, *n* = 100, γ_kin_ = 0. 5, γ_off_ = 0. 5, γ_R_ = 0. 7, κ = 1, m_T_ = 10, r_T_ = 10, α = 1. Changed: γ_R_.

**Figure 4:**
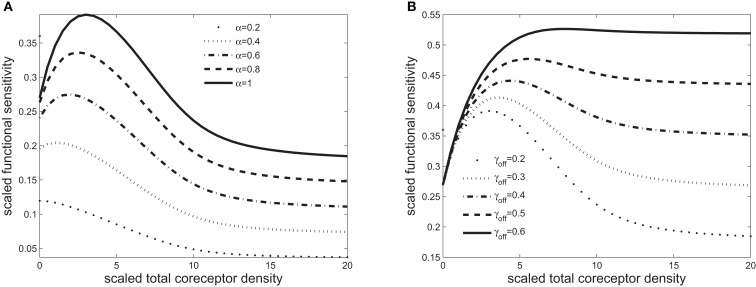
**Scaled functional sensitivity *w* as a function of scaled total CD8 density *x_T_***. **(A)** Curves for various values of the scaled TCR/pMHCI off-rate *α*. **(B)** Curves for various values of the dimensionless factor γ_off_ expressing the modulatory effect of CD8 on the TCR/pMHCI off-rate. Parameters, unless stated otherwise, are as follows: δ = 500, *n* = 100, γ_kin_ = 0. 2, γ_off_ = 0. 2, γ_R_ = 0. 01, κ = 1, *m_T_* = 10, *r_T_* = 10, α = 1. Changed: δ, γ_kin_, γ_R_,α,κ, and *α*’s in **(A)** and γ_off_’s in **(B)**.

**Figure 5:**
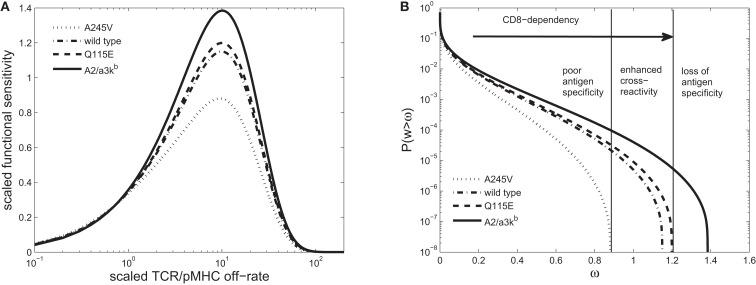
**(A)** Scaled functional sensitivity *w* as a function of scaled TCR/pMHCI off-rate *α*. **(B)** Degeneracy curves ℙ(w > ω) for HLA A*0201 mutants with altered binding affinity for CD8: A245V (dotted line), wild-type (semi-dashed line), Q115E (dashed line), and A2/α3k^b^ (solid line). The three regions represent the overall pattern of CD8^+^ T-cell antigen specificity and the arrow indicates the strength of pMHCI/CD8 interaction. Wild-type parameters are as follows: δ = 0.2, *n* = 100, γ_kin_ = 0. 5, γ_off_ = 0. 5, γ_R_ = 0. 2, κ = 1, *m_T_* = 10, *r_T_* = 10, *x_T_* = 10; the parameter δ is adjusted in proportion to the ratio between the mutant pMHCI/CD8 affinity constant (i.e., dissociation constant) and the wild-type affinity, whereas the parameters κ and *x_T_* are adjusted in inverse proportion to the mutant/wild-type pMHCI/CD8 affinity (these adjustments follow from the scaling). The log-normal distribution has mean 5 and SD 0.5. Figure 5C has been deleted.

The kinetic scheme (Figure 1) and the equations are all unaltered, except for a typographical error in the subscript of a quantity appearing in one of the equations. Specifically, equation (23) in the published text should read:

(2) $$W=M_{R}\lambda_{-1}P_{0}^{0}+M_{XR}\lambda_{-4}P_{0}^{*}.$$

We apologize profusely for any inconvenience our oversight may have caused.

## Conflict of Interest Statement

The authors declare that the research was conducted in the absence of any commercial or financial relationships that could be construed as a potential conflict of interest.

